# Commentary: Evaluating Delivery of a CBT-Based Group Intervention for Schoolchildren With Emotional Problems: Examining the Reliability and Applicability of a Video-Based Adherence and Competence Measure

**DOI:** 10.3389/fpsyg.2021.737095

**Published:** 2021-09-27

**Authors:** Bente Storm Mowatt Haugland, Gro Janne Wergeland

**Affiliations:** ^1^Department of Clinical Psychology, University of Bergen, Bergen, Norway; ^2^Division of Psychiatry, Department of Child and Adolescent Psychiatry, Haukeland University Hospital, Bergen, Norway; ^3^Department of Clinical Medicine, Faculty of Medicine, University of Bergen, Bergen, Norway

**Keywords:** adherence, competence, indicated prevention, youth, anxiety, group CBT

## Evaluating Adherence and Competence in an Indicated Prevention Study

In a recent article by Rasmussen et al. (2021) the reliability and applicability of a measure on treatment adherence and competence in cognitive behavioral therapy (CBT) for youth was examined. The study was part of a Norwegian multi-site randomized controlled trial (RCT) on indicated preventive group-intervention for youth with symptoms of anxiety and depression, using the intervention EMOTION: Kids Coping with Anxiety and Depression. Therapists from community services (e.g., psychologists, school health nurses, counselors, educators) delivered the intervention. The measure applied to evaluate therapist adherence and competence was the Competence and Adherence Scale for CBT (CAS-CBT; Bjaastad et al., 2016).

CAS-CBT is an 11-item observation-based scale designed to assess adherence and competence in delivering CBT for youth with anxiety. The measure assesses CBT structure, process, and relational skills, in addition to the two main goals of each session in the intervention. Psychometric qualities of the scale have been examined previously in clinical samples (e.g., Bjaastad et al., 2016; Harstad et al., 2021). The scoring of CAS-CBT is usually based on video-recordings of therapy sessions, which was also the case in the Rasmussen et al. study.

After investigating the reliability of CAS-CBT, Rasmussen et al. (2021) found the scale to be useful, although they also identified limitations and suggested improvements in the measure. We find the Rasmussen et al. (2021) study to be well-conducted and have no objections regarding their conclusions. Their discussion about the need to consider treatment fidelity in youth mental health and the emphasis on examining treatment fidelity with therapists delivering indicated prevention is particularly important.

We are pleased that the CAS-CBT measure was used and that other research groups are evaluating the scale—the first author of this commentary is co-author of the CAS- CBT. However, in their article Rasmussen et al. (2021) argued that this was the first study to apply CAS-CBT in group CBT within a prevention setting, and with therapists not working in regular clinical practice delivering the intervention. They stated that besides their own study only three studies had used CAS-CBT to evaluate therapist adherence and competence—and these have all been conducted within clinical settings. Finally, they emphasized the need for research on adherence and competence in interventions within the preventive field—arguing that youth recruited to prevention studies are different from youth involved in clinical trials. They claimed that resources to support implementation in community settings are often more limited compared to clinical settings, and that assessments of adherence and competence therefore often are omitted from prevention studies.

## Missing a Previous Indicated Prevention Study Applying CAS-CBT

Regretfully, Rasmussen and colleagues failed to recognize a previous large RCT where CAS-CBT was used to assess adherence and competence in indicated preventive CBT for youth anxiety delivered in group format (see Haugland et al., 2017, 2020; Husabo et al., 2021). In this RCT the CBT was delivered primarily by community providers employed by school health services or primary health services (e.g., school health nurses, community psychologists). Although the participants in the Haugland et al. study 2020 were somewhat older (mean age 14.0 years, SD = 0.84) compared to the Rasmussen et al. (2021) study (mean age 10.1, SD 0.90), both studies were conducted within the same context and cultural setting (i.e., in schools in Norway).

There are interesting differences in methods between Rasmussen et al. (2021) and Haugland et al. (2020). These differences include student raters in Rasmussen et al. (2021) vs. experienced CBT therapists, including the CAS-CBT developers, in Haugland et al. (2020); recording of 20% of the sessions in the Rasmussen et al. study vs. recording of all sessions in the Haugland et al. study; and differences in sessions between the interventions (an extensive 20 session program in Rasmussen et al., 2021 vs. a brief 5-session and a standard 10-session program in Haugland et al., 2020). These differences could have broadened the discussion of the findings in Rasmussen et al. (2020). For example, Haugland et al. (2020) reported higher levels of adherence and competence for the brief compared to the longer program, suggesting that it is easier for novice CBT providers to achieve fidelity in simplified and less flexible interventions (Husabo et al., 2021). This is supporting a point made by Rasmussen et al. that EMOTION is a comprehensive intervention, and that this could have had an impact on program fidelity.

Both studies found CAS-CBT to be a useful measure for settings outside clinical treatment. However, Rasmussen et al. (2020) concluded that low agreement between raters, particularly for process and relational skills, was a limitation of CAS-CBT in this setting. Contrary to this average good agreement between raters was found for adherence [ICC (2,1) = 0.63] and competence [ICC (2,1) = 0.69] in the Haugland et al. (2020) study. These similarities and differences should have been discussed in view of differences in rater background, extensiveness of the programs, and statistical model chosen for the ICC. We believe that additional information from the previous study using the same instrument in a similar context (i.e., indicative prevention, group format, primary health setting) should have been included in the discussion of the findings in the Rasmussen et al. (2021) study.

## Conclusion

The findings of the Rasmussen et al. study 2020 on reliability and applicability of CAS-CBT are scientifically important. However, their statement that this is the first study using CAS-CBT within an indicated prevention study is incorrect. Furthermore, a comparison with findings from a large previous RCT on indicated prevention for youth with anxiety, recruited and delivered within the same cultural context, would have broadened, and probably nuanced the discussion of the findings in Rasmussen et al. (2021) study.

## Author Contributions

BSMH and GJW wrote and edited the commentary.

## Funding

The RCT-study was supported by The Research Council of Norway [Grant no. 229020]. Additional financial support was received from the Oslofjord fund [Grant no. 245807], Regional Research fund western Norway [Grant no. 235707], and the Norwegian Directorate of Health [Reference no. 11/751-38 and 14/4285-3].

## Conflict of Interest

The authors declare that the research was conducted in the absence of any commercial or financial relationships that could be construed as a potential conflict of interest.

## Publisher's Note

All claims expressed in this article are solely those of the authors and do not necessarily represent those of their affiliated organizations, or those of the publisher, the editors and the reviewers. Any product that may be evaluated in this article, or claim that may be made by its manufacturer, is not guaranteed or endorsed by the publisher.
